# Marine Polysaccharides Modulating the Gut Microbiota-Immune Axis in Digestive Tract Tumors: An Update

**DOI:** 10.3390/md24050148

**Published:** 2026-04-23

**Authors:** Lisheng Wang, Danni Gao, Xi Chen, Yitao Chen

**Affiliations:** Jinhua Academy of Zhejiang Chinese Medical University, Jinhua 321000, China; 202531124011089@zcmu.edu.cn (L.W.); gaodanni@zhezhonglab.ac.cn (D.G.); chenxi@zhezhonglab.ac.cn (X.C.)

**Keywords:** marine polysaccharides, gut microbiota–immune axis, digestive tract tumors, immunomodulation, tumor microenvironment, structure–activity relationship, nanodelivery

## Abstract

Digestive tract tumors represent a predominant contributor to the global public health burden, with conventional therapeutic modalities experiencing inherent limitations and immunotherapy being impeded by the immunosuppressive property and heterogeneity of the tumor microenvironment (TME). This makes the gut microbiota–immune axis a promising therapeutic target. Marine polysaccharides, endowed with distinctive structural characteristics, exhibit potential in the modulation of this regulatory axis, yet their structure–activity relationships (SARs) and the intrinsic limitations in delivery efficiency remain largely unelucidated. In this review, we systematically synthesized the latest research advances pertaining to the modulation of the gut microbiota–immune axis by marine polysaccharides in digestive tract tumors, in accordance with the logical framework of polysaccharide structure, flora regulation, immune activation, tumor inhibition, and delivery optimization. We elaborated on the bidirectional crosstalk between the gut microbiota and the immune axis during tumorigenesis, as well as the regulatory effects and core underlying mechanisms of marine polysaccharides derived from algal, animal and microbial sources on this axis, including targeted floral regulation, microbiota-mediated immune activation, and direct/indirect tumor suppression. We also analyzed the key structural determinants and structural modification strategies of marine polysaccharides, alongside the development of nanodelivery systems for the improvement of their oral bioavailability. Furthermore, we identified critical existing research gaps, such as the ambiguous SARs and poor oral bioavailability of marine polysaccharides, and propose the integration of multi-omics analysis, synthetic biology technology and advanced nanodelivery strategies as the core future research directions in this field. Collectively, marine polysaccharides hold tremendous promise as novel therapeutic agents for digestive tract tumors, and interdisciplinary collaboration is regarded as indispensable for their successful clinical translation and translational application.

## 1. Introduction

Digestive tract malignancies represent a leading global cause of disease burden and cancer-related mortality, thus imposing an immense public health challenge worldwide. In 2018, approximately 4.8 million new cases and 3.4 million cancer-related deaths from digestive tract malignancies were reported worldwide, accounting for 26% of newly diagnosed cancer cases and 35% of all cancer-related deaths globally; this disease burden is continuously escalating alongside the global population aging [1]. By 2022, five major gastrointestinal and hepatopancreatic malignancies (gastric, colorectal, hepatic, esophageal, and pancreatic cancer) resulted in approximately 4.783 million new diagnoses and 3.236 million cancer-related deaths globally [2,3]. China bears one of the heaviest burdens of gastrointestinal and liver diseases (GILDs) worldwide; from 1990 to 2019, although the incidence of most GILDs exhibited a declining trend, the disease burden of colorectal and pancreatic cancer increased markedly, with gastrointestinal and hepatic malignancies remaining the leading causes of cancer-related morbidity and mortality in the country [4,5]. Current clinical management of digestive tract malignancies adopts a multidisciplinary collaborative model; however, conventional therapeutic approaches are characterized by inherent clinical limitations [6]. Immunotherapy has emerged as a promising therapeutic strategy for these malignancies, yet it necessitates personalized implementation, and immune checkpoint inhibitors, as the core of immunotherapeutic regimens, carry a potential risk of inducing autoimmune adverse events [7]. Combined immunochemotherapy regimens may yield improved clinical outcomes in gastric cancer treatment; yet their clinical benefits remain unvalidated, and such combination strategies may also increase the risk of treatment-related adverse events, which is largely attributed to the immunosuppressive tumor microenvironment (TME) and its pronounced heterogeneity [8].

The intestinal tract constitutes a dynamic homeostatic ecosystem co-regulated by the gut microbiota and the host immune system, where the regional distribution of intestinal microorganisms and immune cells sustains intestinal immune homeostasis and modulates inflammatory responses to maintain overall intestinal physiological balance [9]. During the progression of digestive tract malignancies, the gut microbiota enriched with pathogenic bacteria facilitates tumor immune escape and impairs the host’s anti-tumor immunity, whereas beneficial commensal bacteria act as potential cancer probiotics or immunoadjuvants in anti-tumor therapy [10]. Intrinsically, intestinal microorganisms exert a regulatory effect on the biological function of phagocytes; specifically, beneficial commensal bacteria stimulate macrophages to secrete a variety of cytokines, which in turn promote the differentiation of regulatory T cells (Tregs) and inhibit the activation of T helper 17 cells (Th17), thereby contributing to the maintenance of intestinal immune tolerance [11]. Invasion by intestinal pathogenic bacteria activates NOD-like receptor family CARD domain-containing protein 4 (NLRC4)-dependent inflammasomes in phagocytes, which subsequently induce the secretion of interleukin-1β (IL-1β) and elicit the host’s protective innate immune response [11]. The gut microbiota–immune axis has therefore emerged as a novel and promising therapeutic target for digestive tract malignancies, which underscores the urgent clinical need for safe and effective targeted modulatory agents against this axis.

Marine polysaccharides, characterized by diverse natural sources and distinctive structural features, have attracted increasing research attention for their potential applications in cancer therapy and immunomodulation. These natural polysaccharides are primarily extracted from macroalgae, marine invertebrates, and deep-sea microbial strains [12]. Unlike terrestrial polysaccharides, marine polysaccharides display a higher degree of sulfation, and their structural characteristics exhibit significant intersource heterogeneity [12]. For instance, fucoidan derived from brown algae features a fucose-based main chain with α-(1 → 3)/(1 → 4) glycosidic linkages and sulfate group modifications, while sea cucumbers contain unique sulfated polysaccharides (SPs) such as fucosylated chondroitin sulfate and sulfated fucoidan (SF) [13,14]. A branched glucomannan is produced by the deep-sea strain *Halomonas* sp. 2E1 [15], and chemical modification enhances bioactivity [16]. Sulfation patterns, molecular weight (MW), and glycan conformation determine bioactivity [17]. Chemical and enzymatic modifications can enhance bioactivity [16].

Marine polysaccharides demonstrate potential in modulating the gut microbiota–immune axis and exerting anti-tumor effects in digestive tract malignancies. For example, fucoidan from brown algae can regulate macrophage polarization, suppress the production of pro-inflammatory cytokines, inhibit leukocyte migration via P-selectin antagonism, modulate MAPK/NF-κB signaling pathways, and ameliorate the tumor immune microenvironment by regulating the composition and functional status of tumor-infiltrating immune cell populations [18,19]. As prebiotics, marine polysaccharides maintain intestinal balance; fucoidan can reverse dysbiosis, restore microbial diversity, and enhance intestinal barrier function in colorectal cancer models [20]. It also exerts direct tumor-inhibitory effects: fucoidan suppresses tumor cell proliferation, induces apoptosis, and causes G0/G1 cell cycle arrest via the inhibition of the Wnt/β-catenin pathway and activation of the Hippo pathway [21,22].

Despite progress, critical gaps exist. Marine polysaccharides enhance immunity via barrier improvement, microbiota remodeling, and TLR4/NF-κB activation [23], but their structure–activity relationships remain unclear, hindering precise design. Orally, their low bioavailability and lack of targeted delivery limit applications [23], which must be addressed for successful clinical translation.

This review synthesizes current research on marine polysaccharides regulating the gut microbiota–immune axis in digestive tract tumors. The content follows a logical progression. It begins with the bidirectional regulatory network of the gut microbiota–immune axis. It then categorizes representative marine polysaccharides by biological source. It elaborates their hierarchical mechanisms of action. It analyzes key structural determinants and modification strategies. It finally addresses nanodelivery systems for bioavailability enhancement. Critical research gaps are identified. These include incomplete structure–activity relationships, poor oral bioavailability, and limited clinical validation. Future priorities are proposed that integrate multi-omics analysis should be, where synthetic biology should enable precise structural optimization, and advanced nanodelivery strategies should improve targeting. This work highlights the therapeutic potential of marine polysaccharides. It provides mechanistic insights and offers translational guidance. The content remains accessible to non-specialists.

## 2. Regulatory Network of the Gut Microbiota–Immune Axis Associated with Digestive Tract Tumors

**Figure 1:**
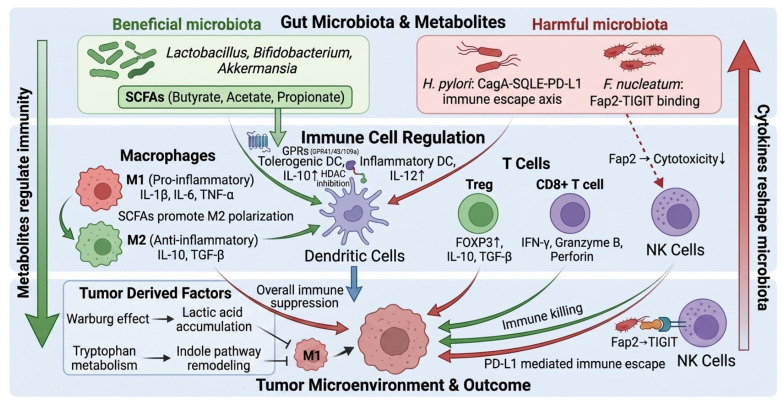
Bidirectional crosstalk between gut microbiota and immune cells in digestive tract tumors. Beneficial bacteria produce SCFAs that promote anti-inflammatory M2 macrophage polarization and Treg differentiation via GPR109a and HDAC inhibition. Pathogenic bacteria drive immune escape through CagA-mediated PD-L1 upregulation and Fap2-mediated TIGIT binding. Tumor-derived factors further reshape microbiota composition and promote immunosuppression.

The gut microbiota–immune axis is a dynamic regulatory network critical to digestive tract tumor progression. This bidirectional crosstalk involves two processes. First, gut microbiota and their metabolites modulate tumor immunity through immune cell polarization, cytokine secretion, and checkpoint expression. Conversely, the digestive tract TME, characterized by metabolic reprogramming and immunosuppression, reshapes microbial composition and function (see Figure 1). Understanding this reciprocal interaction is key to clarifying how marine polysaccharides exert anti-tumor effects via the axis. The section below elaborates on bidirectional microbiota–tumor immunity regulation and the TME’s remodeling role.

### 2.1. Bidirectional Regulatory Effects of the Gut Microbiota on Tumor Immunity

The gut microbiota exerts dual effects on tumor immunity through distinct molecular mechanisms. Beneficial commensal bacteria activate anti-tumor immunity, while pathogenic bacteria promote immune evasion and tumor progression.

Beneficial flora and immune activation. Beneficial commensals (*Lactobacillus*, *Bifidobacterium*) enhance anti-tumor immunity via metabolites, particularly SCFAs and polysaccharide A [24,25,26,27]. SCFAs function through dual signaling mechanisms. They activate G protein-coupled receptors (GPRs), including GPR41, GPR43, and GPR109a on immune cell surfaces [24,28]. Simultaneously, SCFAs enter cells and inhibit HDACs, thereby promoting anti-inflammatory gene expression [29].

These molecular actions lead to coordinated activation of multiple immune cell types. Dendritic cells (DCs) undergo maturation and acquire enhanced antigen-presenting capacity [30]. Macrophages polarize toward the pro-inflammatory M1 phenotype, characterized by increased phagocytic activity and cytokine production [31]. CD8^+^ T cells maintain their memory and effector functions, enabling sustained cytotoxic responses against tumor cells [25,26]. Additionally, SCFAs suppress pro-inflammatory cytokines, including tumor necrosis factor-α (TNF-α) and IL-6 through NF-κB pathway inhibition, thereby attenuating chronic inflammation that would otherwise promote tumor development [27].

Harmful flora and immune suppression. In contrast to beneficial commensals, pathogenic bacteria actively suppress anti-tumor immunity through distinct virulence factor-mediated mechanisms. *Helicobacter pylori* (*H. pylori*) and *Fusobacterium nucleatum* represent two well-characterized examples of tumor-promoting pathogens [32,33].

*H. pylori* induces immune tolerance through its cytotoxin-associated gene A (CagA) protein [32,34]. The CagA protein is delivered into host cells via the type IV secretion system [32]. Upon entry, CagA activates RAS/ERK and PI3K/AKT signaling [32]. This upregulates squalene epoxidase, a key enzyme for cholesterol biosynthesis. Increased cholesterol then promotes PD-L1 palmitoylation and stabilizes its surface expression. Stabilized PD-L1 binds to T cell PD-1, inducing exhaustion and attenuating anti-tumor immunity [34,35]. This sequence constitutes the CagA-SQLE-PD-L1 immune escape axis [34,35]. Concurrently, CagA synergizes with pro-inflammatory signaling through c-Met/PI3K/AKT, TRAF6/TAK1, and NF-κB pathways to induce IL-1β and IL-8, establishing a chronic inflammatory microenvironment that favors tumor progression [32].

*Fusobacterium nucleatum* suppresses natural killer cell (NK) function through *Fusobacterium* adhesin protein 2 (Fap2) [33,36]. The Fap2 protein recognizes and binds to galactose-N-acetyl galactosamine (Gal-GalNAc) glycans overexpressed on tumor cell surfaces [33]. This interaction mediates bacterial enrichment in colorectal and breast tumor tissues [33]. As an immunomodulatory molecule, Fap2 directly engages TIGIT on NK cells [36,37]. This binding transmits inhibitory signals through an immunoreceptor tyrosine-based inhibitory motif and immunoglobulin tail-tyrosine motif-like domains (ITT) within the TIGIT intracellular region [36,37]. The resulting signal transduction weakens NK cell cytotoxicity against tumor targets, thereby impairing anti-tumor immunity [36,37].

### 2.2. Reverse Remodeling of the Microbiota–Immune Axis by the Digestive Tract TME

Tumor cells generate large amounts of lactic acid via the Warburg effect, endowing the TME with high lactic acid concentration and acidic characteristics. This high lactic acid microenvironment can directly inhibit the proliferation and function of effector T cells and limit their utilization of key nutrients such as glucose and glutamine through metabolic competition, thereby weakening the anti-tumor immune response. In contrast, Tregs have stronger adaptability to the low-nutrient and high-lactic acid environment, further exacerbating the immunosuppressive state [38]. In addition, lactic acid is not as simple as metabolic waste; it can act as a circulating carbon source and signaling molecule, participate in immune regulation and barrier homeostasis maintenance through receptors such as GPR81, and affect immune function at both local and systemic levels [39,40]. Beyond its direct effects on the TME, tumor-derived lactic acid can also alter the local intestinal metabolic state and pH environment, thereby affecting the functions of mucosa-associated immune cells and the ecological balance of the gut microbiota.

In addition to lactic acid, tryptophan metabolism links tumor metabolism to intestinal dysbiosis. This process particularly involves indole pathway remodeling. In patients with colorectal cancer, the indole/tryptophan ratio decreases, while the levels of metabolites such as indole-3-aldehyde and indole-3-carboxylic acid increase, and beneficial bacteria with indole-producing ability are significantly reduced [41,42]. Under stress or tumor conditions, indole metabolite intermediates impair epithelial differentiation and barrier function. They also weaken beneficial bacteria’s competitiveness and promote *Fusobacterium nucleatum* enrichment [43].

As a central player in immunosuppression, Tregs function as the core immunosuppressive cells in the TME and collectively attenuate anti-tumor immunity through diverse mechanisms. Tregs can secrete inhibitory cytokines such as transforming growth factor-β (TGF-β), IL-10, and IL-35 to directly inhibit the functions of CD8^+^ T cells and DCs. They also induce T cell exhaustion and immune tolerance through three mechanisms. These include IL-2 competitive consumption, CD39/CD73-adenosine pathway activation, and co-stimulatory signal inhibition via CTLA-4 and LAG3 [44]. Among these, IL-35 can synergistically upregulate the expression of inhibitory receptors such as PD-1 and T cell immunoglobulin and mucin-domain-containing-3 (TIM3) through the B lymphocyte-induced maturation protein 1 (BLIMP1) transcriptional axis, further enhancing the immunosuppressive state [45,46]. In microbiota-associated tumors, a tight immune regulatory network is formed between Tregs and macrophages: bacteria such as *H. pylori* and *Fusobacterium nucleatum* can affect the activation state of macrophages through TLR/NF-κB signals and induce their polarization towards the immunosuppressive M2 phenotype [47,48,49].

Building on the above cytokine network, TGF-β inhibits DC antigen presentation and MHC-II expression. It also induces immunosuppressive molecules, including indoleamine 2,3-dioxygenase (IDO). Furthermore, it directly suppresses the expression of NK cell-activating receptors and interferon-γ (IFN-γ) production. At the same time, TGF-β promotes the differentiation and expansion of Tregs, drives macrophage polarization toward an M2 phenotype, and inhibits NF-κB-mediated inflammatory responses through SMAD6/7 signaling, collectively establishing a profoundly immunosuppressive TME [50,51].

Digestive tract tumor progression follows a self-reinforcing cascade. Tumor metabolic reprogramming driven by the Warburg effect leads to progressive lactate accumulation. Concurrently, microbiota dysbiosis emerges, characterized by indole pathway disruption and enrichment of *Fusobacterium nucleatum*. Simultaneously, immune cells undergo systematic remodeling: Tregs expand, macrophages polarize toward the M2 phenotype, and T cells enter exhaustion. These three processes do not operate in isolation; they fuel one another in a vicious cycle. Consequently, interventions targeting any single node prove insufficient. This complex regulatory network offers multiple intervention targets for marine polysaccharides. Section 3 categorizes representative marine polysaccharides by biological source and outlines their functional profiles, while Section 4 systematically elucidates their hierarchical mechanisms of action.

## 3. Representative Marine Polysaccharides Regulating the Gut Microbiota–Immune Axis in Digestive Tract Tumors

**Figure 2:**
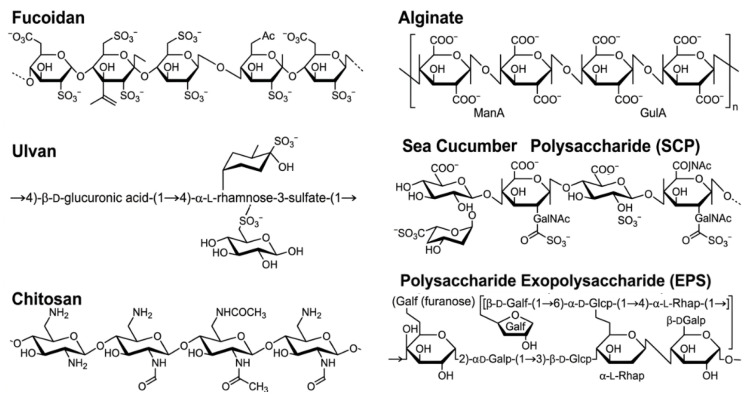
Representative chemical structures of marine polysaccharides derived from different sources.

**Figure 3:**
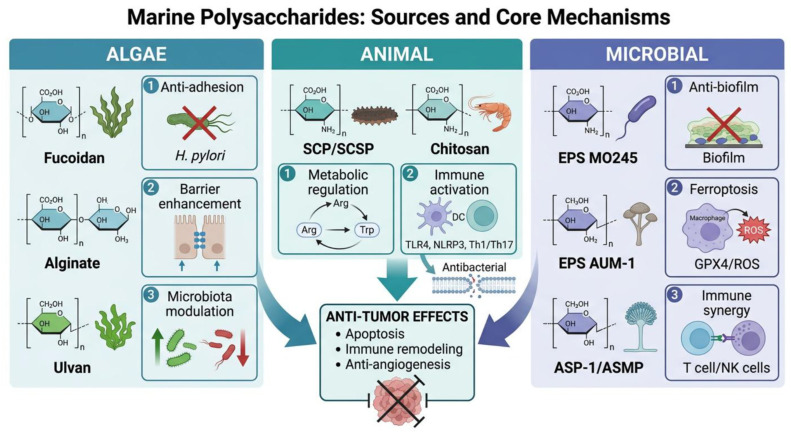
Representative marine polysaccharides and their core mechanisms. Algae-derived polysaccharides exhibit anti-adhesion, barrier enhancement, and microbiota modulation effects. Animal-derived polysaccharides regulate metabolic pathways and activate immune responses. Microbial EPSs display anti-biofilm activity, ferroptosis regulation, and immune–metabolic synergy.

Marine polysaccharides, particularly SPs from algae, marine animals and microorganisms, are potent modulators of the gut microbiota–immune axis in digestive tract tumors. As prebiotics, these non-digestible carbohydrates selectively stimulate the growth and/or activity of beneficial gut microbiota, conferring health benefits to the host. As outlined in Section 2, representative chemical structures of the marine polysaccharides mentioned in this section are shown in Figure 2. These polysaccharides intervene at four key nodes: microbial composition regulation, intestinal barrier strengthening, metabolic modulation, and immune signaling activation (Figure 3). Table 1 summarizes the sources, structural characteristics, and functional profiles of representative marine polysaccharides. The following sections classify these by biological source; their detailed molecular mechanisms are systematically elaborated in Section 4.

### 3.1. Algae-Derived Polysaccharides

Fucoidan, a major SPs component in the cell walls and intercellular matrix of brown algae, exerts anti-infective activity by directly inhibiting the adhesion and colonization of *H. pylori* in the gastric mucosa. [52]. As a prebiotic substrate, fucoidan resists digestion in the upper gastrointestinal tract and reaches the colon intact, where it is selectively fermented by beneficial bacteria such as *Bifidobacterium* and *Lactobacillus*, promoting their proliferation and enhancing SCFA production [52]. Experimental evidence confirms two mechanisms: first, fucoidan binds to gastric mucosal mucins; second, it selectively interacts with fucose-binding proteins on the surface of *H. pylori*. Both actions block bacterial adhesion factors from engaging host cell receptors. This molecular interaction inhibits the initial adhesion of *H. pylori* to gastric epithelial cells and reduces the rate of bacterial infection, which is reflected in in vivo models by a decreased abundance of colonizing bacteria in the gastric mucosa and reduced levels of infection-related markers, including urease and CagA-immunoglobulin G (IgG) [53,54]. Beyond direct anti-adhesion effects, fucoidan enhances host defense through immunomodulation, primarily via pattern recognition receptor (PRR) signaling (see Section 4.2 for detailed mechanisms) [79,80,81,82].

Alginate, a natural polysaccharide constituent of brown algal cell walls, serves as a structural matrix for these marine organisms, and its oligosaccharide derivatives act as selective metabolic substrates for intestinal flora. The prebiotic potential of alginate oligosaccharides lies in their selective utilization by beneficial gut bacteria, particularly *Bacteroides* and *Lactobacillus* species, which possess specific glycoside hydrolases capable of degrading these polymers [55,56]. This selective fermentation induces an increase in the production of SCFAs in the gut. At the level of the intestinal physical barrier, alginate oligosaccharides upregulate the expression of tight junction proteins, increase the number of goblet cells in the intestinal epithelium, and reduce the rate of epithelial cell apoptosis, thereby decreasing intestinal permeability, inhibiting endotoxin translocation, and maintaining the stability of the mucosal barrier [56]. In terms of immunomodulation, alginate oligosaccharides construct a favorable microenvironmental niche for the activation of NK cells. In contrast, high-molecular-weight (HMW) brown algal polysaccharides directly promote NK cell proliferation in the spleen and peripheral blood. They also enhance granzyme B and IFN-γ secretion by these effector cells, thereby improving cytotoxicity against tumors and viral targets. Low-molecular-weight (LMW) alginate oligosaccharides, while unable to directly enter the systemic circulatory system, can indirectly maintain the homeostasis and functional activity of NK cells by alleviating intestinal inflammation, inhibiting oxidative stress, and preserving the balance of key cytokines such as IL-2 and IL-15 [57].

Similarly, ulvan, a SP extracted from green algae including *Ulva lactuca*, exerts a regulatory effect on the intestinal microecology by promoting the proliferation of beneficial bacterial taxa such as *Akkermansia muciniphila*, *Bifidobacterium*, and *Lactobacillus*. This prebiotic effect is attributed to the unique glycosidic linkages and sulfation patterns of ulvan that serve as specific carbon sources for beneficial microbes while inhibiting pathogen growth [58]. This polysaccharide also increases the abundance of SCFA-producing bacteria such as *Bacteroides* and inhibits the growth of intestinal inflammation-associated flora, including *Desulfovibrio*, thereby ameliorating the balance of the intestinal microecosystem and establishing an anti-inflammatory metabolic microenvironment in the gut [58]. Ulvan indirectly modulates host immune function by remodeling intestinal flora composition and regulating metabolic output. Specifically, microbial metabolites such as SCFAs regulate macrophage and T lymphocyte activity. They promote anti-inflammatory cytokine secretion and suppress pro-inflammatory factors. This immunomodulatory profile confers protective effects in experimental models of inflammatory bowel disease (IBD) and other intestinal inflammatory disorders [59,60].

### 3.2. Animal-Derived Polysaccharides

Sea cucumber polysaccharides (SCPs) and their SPs can optimize the intestinal flora structure to regulate the host’s arginine metabolic pathway. The prebiotic activity of SCP is evidenced by its selective promotion of beneficial genera, including *Bacteroides* and *Lactobacillus*, coupled with the suppression of opportunistic pathogens such as *Enterococcus* and *Desulfovibrio* [61,62]. After SCP intervention, the contents of citrulline and L-arginine, key metabolites in the arginine biosynthesis pathway, increase, accompanied by the upregulated expression of related genes [61,62]. After the intervention of sea cucumbers with sulfated polysaccharides, amino acid metabolites related to immunomodulation are also significantly upregulated [63], and this may also indirectly regulate the balance of amino acid metabolic pathways such as arginine by affecting the cross-regulation of sulfur metabolism and nitrogen metabolism [64]. Through the regulation of the arginine metabolic pathway, SCP effectively prevents the excessive activation of immune responses. Arginine metabolism enhances immune function through nitric oxide (NO) synthesis and regulation of innate/adaptive responses. Conversely, SCP modulates the flora–metabolism–transcriptome network. It affects tryptophan metabolism via the IDO1/Kyn/AHR axis, thereby preventing immunosuppressive microenvironment formation [14,65].

In parallel, chitosan and its derivatives exert their antibacterial and immunomodulatory functions in the intestine. Chitosan oligosaccharides demonstrate prebiotic properties by enhancing the growth of *Lactobacillus* and *Bifidobacterium* while modulating the *Firmicutes*/*Bacteroidetes* ratio, thereby improving microbial diversity and gut homeostasis. Because chitosan’s polycationic properties enable electrostatic interactions with negatively charged bacterial membrane structures, this causes membrane damage, content leakage, and cell death. In addition, chitosan forms a dense polymer membrane on bacterial surfaces. This covers Gram-negative outer membrane pores, blocks nutrient exchange, and inhibits proliferation [66,67]. Furthermore, chitosan can also interact with signaling pathways such as TLR4 and the cyclic GMP-AMP synthase-stimulator of interferon genes (cGAS-STING), activate the NOD-like receptor family, pyrin-domain-containing-3 (NLRP3) inflammasome, induce type I interferon response, and further enhance the coordination of immune responses. Especially when combined with the Toll-like receptor 9 (TLR9) agonist cytosine–phosphate–guanosine (CpG), chitosan can synergistically promote DCs to secrete cytokines such as IL-12, IL-6, and IL-23, inducing a stronger Th1/Th17 immune response [68,69].

### 3.3. Microorganism-Derived Polysaccharides

Marine bacterial polysaccharide exopolysaccharides (EPSs) represented by MO245 exhibit anti-pathogenic adhesion and anti-biofilm activity independent of bactericidal effects, which inhibits initial adhesion through physicochemical surface modification mechanisms. MO245 can effectively inhibit the adhesion of *Pseudomonas aeruginosa* and *Vibrio harveyi*, and its mechanism lies in adsorbing on the surface to form a hydrophilic barrier and reducing surface hydrophobicity [70]. Similarly, EPSs from marine *Bacillus* and marine *Pseudomonas* can also interfere with the adhesion and biofilm formation of various pathogenic bacteria by changing bacterial surface properties or directly acting as dispersants. Such interventions effectively reduce the colonization advantage of pathogenic bacteria in the local microenvironment [71,72]. AUM-1, an EPS from *Aureobasidium melanogenum*, regulates glutathione peroxidase 4 (GPX4) expression and glutathione (GSH) redox status. By modulating glutamate metabolism and the tricarboxylic acid (TCA) cycle, it regulates the macrophage ferroptosis-related immune processes, collectively improving the immunosuppressive microenvironment [73,74]. Additionally, certain marine bacterial EPSs exhibit prebiotic potential by serving as fermentable substrates for beneficial gut microbiota, promoting the enrichment of SCFA-producing communities and enhancing intestinal barrier integrity [71,72].

In line with the immunomodulatory and metabolic regulatory roles of fungal-derived polysaccharides, metabolomic analysis shows that the EPS ASP-1 isolated from the coral symbiotic fungus *Aspergillus pseudoglaucus* SCAU265 can enhance immune cell function by regulating amino acid metabolism. This immune activation mechanism may indirectly affect the activation and functional status of T cells and NK cells, providing a basis for combined application with PD-1/PD-L1 inhibitors to synergistically enhance anti-tumor immunity [75,76]. The EPS ASP-1 also demonstrates prebiotic characteristics by modulating amino acid metabolism and supporting the growth of commensal bacteria that contribute to host immune homeostasis [75,76].

Additionally, ASMP, a novel polysaccharide from the marine fungus *Aspergillus medius*, enhances cyclooxygenase-2 (COX-2) and inducible nitric oxide synthase (iNOS) expression. Through the Nrf2/SLC7A11/GPX4 axis, it reduces the GSH/GSSG ratio, upregulates ROS, and activates ACSL4, ultimately inducing ferroptosis [77,78].

## 4. Representative Core Mechanisms of Marine Polysaccharides Regulating the Gut Microbiota-Immune Axis

Marine polysaccharides regulate the gut microbiota–immune axis in digestive tract tumors through a hierarchical chain. Upstream microbial remodeling generates metabolic signals that drive midstream immune activation. This collectively establishes an anti-tumor microenvironment, enabling downstream tumor suppression. This progressive mechanism transforms polysaccharide-mediated microbiota modulation into measurable anti-tumor outcomes. Key features, including core pathways, key mediators and regulatory effects, are summarized in Table 2.

### 4.1. Upstream Mechanism: Directional Regulation of Intestinal Microbiota by Marine Polysaccharides

Marine-derived polysaccharides and their derivatives can specifically regulate the composition and proportion of intestinal microorganisms. This regulatory effect is fundamentally linked to their prebiotic properties. Prebiotics are defined as non-digestible food components that beneficially affect host health by selectively stimulating the growth and/or activity of one or a limited number of bacteria in the colon [78]. Marine chitooligosaccharides restore gut microbiota diversity in colitis models, regulate the abnormal *Firmicutes*/*Bacteroidetes* ratio, and increase the relative abundance of beneficial bacterial genera such as *Lactobacillus* [83]. SF from *Laminaria japonica* enriches beneficial genera including *Akkermansia*, *Lactobacillus*, and *Bifidobacterium*. This enrichment correlates with intestinal stem cell marker expression and promotes epithelial development and repair [84].

Resisting upper GI digestion, marine polysaccharides reach the colon intact and serve as selective fermentation substrates for beneficial taxa [85]. This selective fermentation produces SCFAs and other bioactive metabolites that mediate subsequent immune modulation. Marine algal oligosaccharides are fermented by intestinal flora to produce SCFAs. SCFAs not only serve as the main energy source for intestinal epithelial cells but also reduce intestinal pH and inhibit the growth of harmful bacteria [85,86,87].

Besides remodeling the gut microbiota, marine polysaccharides strengthen the intestinal physical barrier through direct and indirect pathways. Chitosan oligosaccharides directly upregulate tight junction protein expression. They also indirectly promote intestinal stem cell proliferation and differentiation through enriched beneficial bacteria. This enhances epithelial barrier regenerative capacity [88]. They also downregulate pro-inflammatory cytokines and inhibit inflammatory signaling activation through flora and metabolite regulation. This exerts protective effects in IBD and other models. This anti-inflammatory effect synergizes with flora regulation and barrier enhancement to form a positive regulatory loop [89,94]. Marine polysaccharides can be combined with probiotics to significantly enhance the adhesion and colonization ability of *Lactobacillus* in the intestine, producing a synergistic effect [56]. This barrier-enhancing effect is synergistically supported by prebiotic-mediated improvements in microbial composition and SCFA production, which promote tight junction protein expression and mucin secretion [89,103].

### 4.2. Midstream Mechanism: Microbiota-Mediated Immune Activation Pathways

Marine polysaccharides modulate the gut microbiota–immune axis through three interconnected mechanisms at the midstream level. These include activation of innate immunity, enhancement of adaptive immunity, and improvement of the inflammatory microenvironment.

Innate immune activation. SCFAs activate innate immune cells through GPR signaling. SCFAs bind to GPR43 and GPR41 receptors on macrophages and DCs, triggering intracellular signaling cascades that promote immune activation [88,89].

In macrophages, engagement with GPR43 and GPR41 drives polarization toward the pro-inflammatory M1 phenotype. M1 macrophages exhibit enhanced phagocytic capacity, increased antigen presentation, and elevated production of ROS and NO [25,94]. This polarization state enables effective clearance of tumor cells and pathogens. Additionally, SCFA-activated macrophages upregulate co-stimulatory molecules such as CD80 and CD86, facilitating T cell priming.

In DCs, SCFA-mediated GPR signaling promotes maturation and enhances the antigen-presenting capacity. Mature DCs migrate to draining lymph nodes, where they activate naive T cells [30]. SCFAs also upregulate MHC-II and co-stimulatory molecule expression on DCs, improving their ability to initiate adaptive immune responses [90].

Adaptive immune enhancement. SCFAs enhance adaptive anti-tumor immunity through multiple coordinated mechanisms. First, SCFAs promote CD8^+^ cytotoxic T cell proliferation and effector function. Through histone deacetylase inhibition and mechanistic targets of rapamycin activation, SCFAs upregulate CD25, IFN-γ, and TNF-α expression in CD8^+^ T cells [91]. These activated cytotoxic T cells efficiently recognize and eliminate tumor cells.

Second, SCFAs suppress Treg differentiation and function. SCFA-mediated histone deacetylase inhibition enhances histone H3 acetylation at the Foxp3 locus, yet the net effect in tumor contexts is suppression of Treg-mediated immune tolerance [29,32]. This occurs through modulation of local cytokine milieu and metabolic competition.

Third, SCFAs downregulate immune checkpoint molecule expression. Both PD-L1 and CTLA-4 expression are attenuated in SCFA-rich microenvironments [34,79]. This reduction in checkpoint molecules restores T cell activity and enhances anti-tumor immune surveillance.

Inflammatory microenvironment improvement. SCFAs reshape the tumor inflammatory microenvironment toward an anti-tumor phenotype through cytokine modulation. SCFAs suppress pro-inflammatory cytokines that promote tumor growth and metastasis. Specifically, TNF-α and IL-6 production are inhibited through NF-κB pathway suppression [92,93]. These cytokines otherwise drive tumor proliferation, angiogenesis, and immune evasion.

Concurrently, SCFAs upregulate anti-tumor and immunostimulatory cytokines. IL-12 production by antigen-presenting cells is enhanced, promoting Th1 differentiation and NK cell activation [29,94]. IFN-γ levels increase, further amplifying anti-tumor immunity and enhancing MHC expression on tumor cells [95,96].

This cytokine reprogramming transforms the TME from immunosuppressive to immunostimulatory. The combination of reduced pro-inflammatory factors and elevated anti-tumor cytokines creates favorable conditions for effective immune-mediated tumor control.

### 4.3. Downstream Mechanism: Direct/Indirect Inhibition of Digestive Tract Tumor Cells

Fucoidan can directly induce tumor cell apoptosis and inhibit their proliferation through multiple mechanisms. Studies have confirmed that fucoidan can reduce the survival rate of tumor cells in a dose- and time-dependent manner, and trigger typical apoptotic characteristics, such as internucleosomal DNA fragmentation, chromatin condensation, activation of caspase-7/8/9, and cleavage of the poly-ADP-ribose polymerase (PARP) protein. The key mechanism activates caspase-8. This cleaves the Bid protein, promoting Bax translocation to the mitochondria. Cytochrome c release follows, ultimately activating caspase-9 and amplifying the apoptotic signal. This process can be completely blocked by a caspase-8-specific inhibitor, indicating that its apoptotic pathway is mainly dependent on caspase-8 [97]. Similarly, fucoidan inhibits proliferation and induces apoptosis in HS-sultan lymphoma cells in a dose-dependent manner. This involves mitochondrial pathway activation and downregulation of ERK and GSK phosphorylation in pro-survival signaling [98]. In addition, marine bacterial capsular polysaccharides can synergistically activate the mitochondrial intrinsic pathway and the death receptor extrinsic pathway, accompanied by activation of p53 and p38 MAPK, with no significant toxicity to normal cells [99]. These studies collectively indicate that fucoidan-like substances can directly and effectively inhibit tumor cell survival and induce their apoptosis through multi-target and multi-pathway synergistic effects.

Fucoidan also indirectly inhibits tumor growth through a strong anti-angiogenic effect. In addition, fucoidan can downregulate the expression of the platelet-derived growth factor (PDGF) and interfere with the vascular maturation process. Meanwhile, it inhibits the HIF-1/VEGF signaling axis, blocking hypoxia-driven angiogenesis in the TME [100]. Studies show that natural fucoidan and its oversulfated form (OSF) directly bind to VEGF. This prevents VEGF from binding to VEGFR-2 on endothelial cells. Consequently, downstream pro-angiogenic signal transduction is inhibited. Furthermore, it blocks the VEGFR2/Erk/VEGF signaling pathway, effectively inhibiting endothelial cell lumen formation, migration, and invasion in vitro and in vivo, and ultimately inhibiting tumor angiogenesis and growth [101]. OSF also regulates angiogenesis through the bFGF signaling pathway. It promotes bFGF binding to heparan sulfate on endothelial cells. This enhances bFGF receptor tyrosine phosphorylation and significantly upregulates PAI-1 expression. Increased PAI-1 levels inhibit the degradation of the extracellular matrix, thereby effectively blocking endothelial cell migration and neovascularization [102].

## 5. Structure–Activity Relationships of Marine Polysaccharides: Impact of Structural Features on Regulatory Activity

### 5.1. Correlation Between Core Structural Parameters and Bioactivity

The immunomodulatory efficacy of marine polysaccharides is governed by three primary structural determinants: MW, sulfation pattern, and glycosidic linkage type. These parameters operate through distinct yet interconnected mechanisms to modulate host immunity and gut microbiota composition.

Molecular weight. A non-linear relationship exists between MW and bioactivity, with an optimal window of 5–50 kDa [104,105]. Below this threshold, insufficient epitope density compromises PRR crosslinking [106]; above it, steric hindrance limits receptor accessibility [107]. LMW chitosan below 10 kDa exemplifies this paradigm. Following TLR4-mediated endocytosis, it facilitates endosomal escape and activates MyD88-dependent signaling. This ultimately upregulates COX-2 and MCP-1 [108,109]. LMW polysaccharides also exhibit enhanced antioxidant capacity due to greater exposure of reactive functional groups. In the gut, LMW species are readily fermented by probiotics. HMW counterparts act primarily through physical mechanisms, specifically by increasing luminal viscosity and forming protective barriers [110,111].

Sulfation pattern. The degree and position of sulfate groups critically influence immunomodulatory and antimicrobial functions. High SPs activate macrophages via NF-κB and p38 MAPK pathways, inducing NO and cytokine production [112]. Sea cucumber-derived SFs further enhance NK cell proliferation and cytotoxicity through IFN-γ, perforin, and granzyme B secretion [113]. Sulfation at specific positions dictates microbial selectivity. C2-sulfation generates axial negative charges that electrostatically engage TLR2/TLR4 co-receptors [114], while C4-sulfation shifts glycan conformation from gt to gg rotamers, optimizing binding to bacterial lectins [115,116]. Notably, C2/C4 co-sulfated derivatives exhibit 3- to 5-fold-enhanced selectivity for *Bacteroides* over *Enterococcus* [117].

Glycosidic Linkage. The β-(1→3) linkage represents a conserved immunostimulatory motif. Kelp polysaccharides LJP-11 and LJP-31, both bearing β-(1→3) backbones, activate macrophages through TLR4-mediated MAPK and NF-κB signaling [118,119]. This linkage adopts an extended helical conformation. It presents a hydrophobic groove recognized by Dectin-1 and TLR4/MD-2. This recognition triggers multivalent receptor clustering and downstream ERK1/2 and p38 phosphorylation [120]. Conversely, the compact helical architecture of α-(1→4)-linked polysaccharides sterically occludes these interfaces, attenuating immunostimulatory potency [121].

Fucosylation and microbial interactions. Variations in fucosylation patterns, encompassing linkage type, branching density, and core structure, modulate bacterial lectin recognition and mucosal colonization [122]. Furthermore, uronic acid and sulfate content in fucoidans guide species-specific microbial utilization, thereby shaping community metabolic profiles [123].

Collectively, these structure–function relationships are summarized in Table 3, which systematically correlates marine polysaccharide structural parameters with their microbiota-regulating and immunomodulatory activities.

### 5.2. Optimization of Bioactivity Through Structural Modification

Functional group modification expands the biological functions of polysaccharides by introducing novel chemical moieties. Sulfation significantly enhances the antioxidant and anticoagulant activities of polysaccharides, with the biological effects being closely correlated with the degree of sulfation and substitution sites. Acetylation and phosphorylation improve physicochemical properties, thereby augmenting free radical-scavenging capacity [125,126,127]. Various marine SPs, after undergoing sulfation, carboxymethylation, or phosphorylation modifications, exhibit markedly improved water solubility and bioavailability, as well as enhanced antioxidant, anti-inflammatory, anticoagulant, and anti-tumor activities [128]. In particular, sulfation modification increases the negative charge density, which strengthens electrostatic interactions with cell surface receptors and thereby elevates bioactivity.

In terms of optimizing structural stability and targeting ability, modifications such as carboxymethylation, thiolation, cationization, and oxidation significantly improve the application performance of polysaccharide-based materials. Carboxymethylation endows polysaccharides with superior water solubility and pH responsiveness, while thiolation enhances their mucoadhesive properties. Cationization facilitates the construction of stable nanocomplexes, and oxidation confers antibacterial activity and stimuli-responsive gelation characteristics [129]. Additionally, ionic or covalent crosslinking technologies enhance the structural stability and functional controllability of polysaccharide-based hydrogels and nanoparticles, which also play a significant role in targeted drug delivery systems.

Selective modification serves as a vital tool for elucidating polysaccharide structure–activity relationships. Enzymatic regioselective removal of specific substituents enables precise structural manipulation. For example, the specific removal of 4-O-sulfate groups from SF results in a significant reduction in its anticancer activity [130], highlighting the functional importance of this modification site.

Enzymatic biomodification, utilizing specific glycosidases and sulfatases, allows for the precise regulation of polysaccharide MW and sulfation patterns to improve biological functions. Studies have shown that sulfatases derived from marine bacteria can be used for enzymatic desulfation, achieving precise control over sulfation patterns. During this process, sulfatases often act in synergy with glycosidases to achieve stepwise depolymerization and MW reduction, ultimately generating oligosaccharide fragments that are more readily utilized by microbes or the host, thereby significantly enhancing bioavailability [131].

Enzymatic degradation also contributes to enhanced bioactivity. By reducing MW and exposing more reactive functional groups, enzymatically degraded polysaccharides typically exhibit stronger antioxidant and immunomodulatory capabilities. For instance, enzymatic degradation of algal polysaccharides significantly reduces viscosity. This facilitates diffusion and absorption in biological systems. It also markedly enhances antioxidant activity and macrophage-activating potential [132,133]. Similarly, LMW fragments generated via directed enzymatic hydrolysis of fucoidan exhibit superior intestinal absorption rates and more potent biological effects in terms of anti-tumor and immunomodulatory activities [134,135].

### 5.3. Research Methods and Challenges in Structure–Activity Relationships

A combination of advanced analytical techniques is widely employed to systematically elucidate multi-level polysaccharide structural information. These range from monosaccharide composition to supramolecular conformation. Techniques include NMR spectroscopy, MS, AF4, and SEC, often coupled with hyphenated detection methods [136]. FT-IR enables the rapid identification of functional groups and glycosidic linkage types, while NMR spectroscopy is pivotal for resolving sulfation sites and linkage patterns. GC-MS or LC-MS are used for the analysis of monosaccharide composition and MW distribution, and HPAEC-PAD and GPC/SEC are commonly utilized for the quantification of sugar composition and MW distribution [137].

However, existing structural analysis techniques face significant limitations in distinguishing polysaccharide mixtures with similar molecular sizes. For example, studies of Dendrobium polysaccharides by different laboratories using HPGPC and NMR yielded significantly varied results. Subsequent investigations revealed that the samples were likely starch and mannan mixtures. These co-eluted as a single chromatographic peak due to similar molecular sizes. This led to their misidentification as a homogeneous pure product [138]. This issue illustrates that a single analytical approach is often insufficient to accurately differentiate between true structural heterogeneity and sample mixing effects, thereby impeding the precise elucidation of structure–activity relationships.

To further investigate the relationship between polysaccharide structures and their function in regulating the gut microbiota, researchers have increasingly adopted in vitro microbiota fermentation models, complemented by in vivo animal experiments for functional validation [139,140]. In vitro fermentation systems allow evaluation of degradation behavior in structurally different polysaccharides. These systems also assess effects on specific microbial group enrichment or inhibition under controlled conditions. This minimizes interference from individual differences. Building on this, combined with in vivo models, it is possible to systematically elucidate the intrinsic connections between polysaccharide structural features, molecular conformation, and biological effects.

## 6. Nanodelivery Strategies of Marine Polysaccharides: Technical Pathways to Enhance Regulatory Efficiency

### 6.1. Advantages and Types of Marine Polysaccharide-Based Nanocarriers

Marine polysaccharides exhibit many significant advantages as nanocarriers in tumor immunotherapy. Their excellent biocompatibility and biodegradability enable effective protection of immunotherapeutic agents. These include cytokines, antibodies, and nucleic acids. This protection prevents enzymatic degradation and immune clearance. Consequently, in vivo circulation time is extended, and efficiency of targeted delivery is improved [141,142,143]. Meanwhile, marine polysaccharide molecules are rich in active functional groups. These facilitate structural modification and functional design. They also allow construction of intelligent drug delivery systems. Such systems exhibit pH, redox, or enzyme-responsive properties and specifically target the TME [142,143,144,145]. Among representative materials, alginate is a natural anionic polysaccharide. Its molecular chain consists of β-D-mannuronic acid and α-L-guluronic acid. The guluronic acid block specifically binds divalent cations such as calcium ions. This forms a classic egg box structure and achieves ion-responsive gelation [144,145]. In addition, fucoidan has good biocompatibility and high affinity for P-selectin. This receptor is constitutively expressed in various tumors. It can be significantly induced or translocated to the cell surface under radiation stimulation. This property provides an activatable tumor targeting mechanism for nanocarriers [144,145].

Chitosan–fucoidan composite nanoparticles are polyelectrolyte composite carriers formed by self-assembly based on electrostatic interactions between the sulfate groups of fucoidan and the amino groups of chitosan [146]. By regulating the ratio of the two components and environmental pH, nanoparticles with a particle size of approximately 100–500 nm can be constructed, which exhibit good biocompatibility and pH-responsive drug release characteristics. This system has been successfully applied for the delivery of various small-molecule and biological drugs [146,147,148].

In nanogel systems, alginate-based carriers form a three-dimensional network through ionic crosslinking and hydrogen bonding, providing effective protection for hydrophobic or unstable drugs [138]. For example, inhalable quercetin nanogels based on alginate not only significantly improve drug water solubility and bioavailability but also exhibit excellent performance in antioxidation, anti-inflammation, and pulmonary targeted delivery [149]. In addition, to meet the needs of local treatment for conditions such as peritoneal metastasis, cisplatin-loaded alginate nanogels are further combined with in situ crosslinking hydrogels to form a “nanogel-hydrogel” composite delivery system. This system achieves dual encapsulation and sustained release through the “egg-box model”, significantly extending the retention time of drugs at the target site [150].

Polysaccharide-modified liposomes, as functionalized lipid nanocarriers, improve the stability and delivery efficiency of traditional liposomes by introducing natural polysaccharides on their surface [151,152]. Polysaccharide modification reduces liposome aggregation. Chitosan, sodium alginate, and hyaluronic acid provide electrostatic and steric hindrance effects. These modifications also enable active targeted delivery. They utilize specific interactions with receptors on tumor cell surfaces. Furthermore, these modified liposomes can realize controlled release in response to pH, enzyme, or redox signals in the TME, enhancing the precision and efficacy of drug delivery [151,152,153].

Each nanodelivery platform presents distinct advantages and limitations. Chitosan-fucoidan nanoparticles offer strong mucoadhesion and scalability. However, they are prone to premature payload release under physiological ionic conditions [154]. Alginate nanogels protect labile biomolecular payloads under mild crosslinking conditions. Yet they remain vulnerable to decrosslinking during systemic circulation [155]. Polysaccharide-modified liposomes leverage established lipid nanocarrier technology with added targeting versatility. But surface coating may reduce encapsulation efficiency and increase fabrication complexity [156]. Critically, no single platform fulfills all requirements for ideal delivery. These include gastrointestinal stability, tumor accumulation, triggered intracellular release, and preserved immunostimulatory activity. This gap provides the rationale for hybrid and stimuli-responsive composite architectures.

### 6.2. Design and Application of Targeted Delivery Strategies

The active targeting strategy of marine polysaccharide nanocarriers achieves precise drug delivery by modifying specific receptor ligands on the surface of tumor cells. For example, a pH-responsive copolymer carrier based on chitosan and sodium hyaluronate achieves sustained methotrexate release. It targets colorectal cancer cells by recognizing highly expressed CD44 receptors through hyaluronic acid. This significantly enhances toxicity to Caco-2 cells and induces apoptosis [153].

Alginate serves as an ideal targeting carrier due to its ease of chemical modification. Folic acid-modified alginate nanoparticles can target cancer cells by binding to folate receptors, while glycosylated alginate can target DCs to enhance immune response. The carboxyl and hydroxyl functional groups of alginates also facilitate covalent conjugation with targeting peptides and antibodies, which improves cell selectivity and enhances drug accumulation and therapeutic effects [142,157]. In addition, alginate carriers exhibit stability and lysozyme resistance in the gastric acid environment; a dual pH-responsive hydrogel system based on sodium alginate and sodium carboxymethylcellulose can simultaneously deliver methotrexate and aspirin, providing chemotherapeutic and analgesic effects [158].

Fucoidan is often used for targeting ligand modification due to the high affinity of its sulfate groups for P-selectin, which is highly expressed at tumor sites. LMWF-Lip can achieve efficient targeted delivery in activated vascular endothelial cells through P-selectin-mediated endocytosis and exhibit significant enrichment capacity in inflammatory models [159].

In the design of marine polysaccharide anti-tumor nanocarriers, the passive targeting strategy mainly relies on the enhanced permeability and retention effect (EPR) of tumor tissues. Marine polysaccharides such as chitosan and sodium alginate, with adjustable particle size and surface charge, are easy to construct hydrophilic or near-neutral surface nanocarriers, thereby extending blood circulation time and promoting their passive enrichment in tumor tissues [160].

In the passive targeting design of polysaccharide-based nanocarriers, optimizing particle size and surface properties is a key factor in improving tumor accumulation efficiency. Studies show that controlling chitosan-based nanocarrier particle size within 30 to 200 nm effectively enhances extravasation through tumor vascular endothelium gaps. Meanwhile, surface PEGylation modification reduces reticuloendothelial system clearance. This achieves long circulation and passive tumor targeting [161]. Sodium alginate nanocarriers rely on pH sensitivity and hydrogel-forming ability. They undergo structural changes or aggregation in the acidic TME. This increases local particle size, reduces backflow, extends retention times, and enhances drug accumulation under the EPR effect [162,163]. By regulating physicochemical properties such as the size, morphology, and surface charge of nanocarriers, the limitations of TME heterogeneity on passive targeting efficiency can be reduced.

Active targeting strategies offer superior tumor selectivity and intracellular delivery but are limited by receptor heterogeneity and increased formulation complexity [164]. Passive EPR-based strategies are simpler but fundamentally constrained by EPR heterogeneity across tumor types [164]. Combined active–passive designs represent the most promising approach: PEGylation enables passive tumor accumulation via prolonged circulation, while stimuli-responsive ligand exposure subsequently facilitates active receptor engagement at the tumor site. Marine polysaccharides are particularly suited for such combinatorial platforms, as their multifunctional groups allow simultaneous PEG conjugation and targeting of ligand attachment without complex synthetic chemistry.

### 6.3. Enhancing Effect of Delivery Systems on Microbiota–Immune Regulatory Activity

Oral colon-targeted delivery systems require various physical or chemical strategies. These enhance gastrointestinal stability. They prevent degradation of polysaccharide carriers by gastric acid and digestive enzymes in the upper gastrointestinal tract. Consequently, carriers achieve intact delivery to the colon and effective colonization. Commonly used methods include acid-resistant coating, crosslinking between polysaccharides, chemical modification, and composite coating technology [165,166]. Among them, pectin forms an “egg-box” structure through calcium ion crosslinking, effectively enhancing its enzyme resistance. In addition, constructing a “nano–micron” composite delivery system by encapsulating nanoparticles into microspheres can provide dual physical protection for active ingredients, further improving their targeted release and colonization efficiency in the colonic environment [167]. In probiotic delivery systems, chitosan-coated alginate microcapsules form a dense coating layer. This effectively blocks erosion by gastric acid and digestive enzymes. Meanwhile, their cationic properties enhance electrostatic interaction with the intestinal mucosa. This extends the intestinal retention time and significantly improves colon colonization efficiency [168].

**Figure 4:**
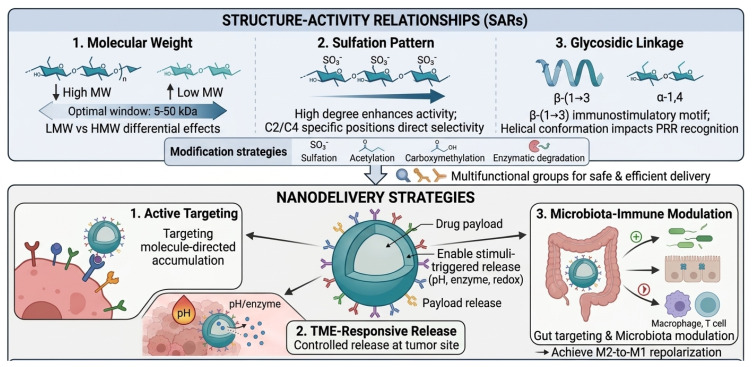
Structure–activity relationships and nanodelivery strategies of marine polysaccharides. Upper panel: Molecular weight, sulfation pattern, and glycosidic linkages determine bioactivity. Lower panel: Polysaccharide-based nanocarriers enable active targeting, TME-responsive release, and microbiota–immune modulation, overcoming limitations of oral bioavailability and targeted delivery.

Polysaccharide-based drug delivery systems can significantly enhance the specific enrichment of carriers at tumor sites and improve anti-tumor immune response through structural modification, targeted design, and TME-responsive mechanisms [141,169]. Polysaccharide-based nanodelivery systems can efficiently encapsulate nucleic acids, immune adjuvants, or antigens, improving their in vivo stability and bioavailability; for example, chitosan-based nanoparticles protect nucleic acids from degradation through electrostatic interaction and achieve controlled release in the TME by virtue of pH-responsive properties [170]. In TME-targeted immunotherapy applications, the carboxymethylated sodium alginate R848 co-loaded nanosystem achieves pH-responsive drug release. Release rates are 2.8-fold higher at TME, mimicking pH 6.5 compared to physiological pH 7.4. This induces measurable M2 to M1 macrophage repolarization. It also produces superior tumor growth inhibition compared to free R848 administration in subcutaneous tumor models [171]. Fucoidan-functionalized doxorubicin nanoparticles demonstrated P-selectin-mediated tumor accumulation. Intratumoral drug concentrations were higher than those achieved with non-targeted doxorubicin formulations. These nanoparticles also uniquely reversed TGF-β-mediated immunosuppression within the TME through fucoidan’s direct receptor interaction. This immunomodulatory effect was absent from non-targeted formulations. It confirms that the polysaccharide component contributes active therapeutic value beyond passive carrier function [171]. Collectively, these findings demonstrate that nanodelivery engineering transforms marine polysaccharides from pharmacokinetically limited bulk materials into precision immunotherapeutic agents with defined targeting mechanisms and measurably improved outcomes. The structure–activity relationships that govern these effects and the advanced nanodelivery strategies designed to optimize them are summarized in Figure 4.

## 7. Conclusions

Structural heterogeneity of polysaccharides is a major challenge in elucidating their mechanisms of action and investigating structure–activity relationships; however, existing analytical methods such as high-performance HPGPC and NMR are insufficient to effectively distinguish polysaccharide mixtures with similar MWs [138]. This structural heterogeneity is closely associated with the polysaccharide source and production process. It aligns with the structural characteristics summarized in Section 3. Different extraction techniques affect MW, monosaccharide composition, and branching structure. These alterations further change interaction patterns with intestinal microbiota and immunomodulatory functions [56,83,172]. Marine-derived polysaccharides such as fucoidan and carrageenan show greater structural variability. Their structures are significantly influenced by species, growth environment, and extraction conditions. This leads to substantial variations in bioactivity and efficacy. Consequently, standardized production and clinical application become more difficult [173,174]. Critically, this structural heterogeneity poses a direct barrier to clinical translation. HMW marine polysaccharides face substantial pharmacokinetic challenges. These include susceptibility to gastric acid and enzymatic degradation, limited intestinal epithelial transport, and microbial modification in the colon. These challenges collectively result in poor systemic bioavailability and unpredictable dose–response relationships [173].

Establishing a more physiologically relevant and standardized research system is necessary to systematically decipher functional mechanisms of complex active substances like polysaccharides. Currently, the humanized microbiota-associated mouse model lacks unified construction standards for evaluating polysaccharide activity. Significant methodological differences exist in donor screening, sample processing, microbiota colonization, and functional verification. These differences limit the comparability and reproducibility of research results [175,176]. This gap directly corresponds to upstream mechanism research relying on intestinal microbiota regulation. It addresses the inconsistency in evaluation methods for the flora-regulating effects of marine polysaccharides. Existing studies have demonstrated that polysaccharides with different structures can regulate immune cell functions through different receptors such as Dectin-1, TLR, and C-type lectin receptor (CLR), as well as signaling pathways including spleen tyrosine kinase (Syk)/NF-κB and MyD88; however, their precise action targets, signal transduction networks, and dynamic processes of interaction with the intestinal microbiota still lack systematic elaboration, which requires in-depth support and integrated analysis of multi-omics technologies [177,178]. This research gap complements the midstream and downstream mechanisms outlined in Section 4, where the microbiota-mediated immune activation pathways and direct/indirect tumor-inhibitory effects have been partially clarified, but the precise regulatory networks linking polysaccharide structure to these mechanisms remain unclear. Clinical evidence for marine polysaccharides in digestive tract tumors remains limited. Fucoidan, the most clinically investigated candidate, has been evaluated primarily in small-scale, open-label trials as an adjunct to chemotherapy, with reported improvements in quality of life and NK cell activity [179], but these studies are constrained by small sample sizes, a lack of randomization, and heterogeneous preparations [180]. No phase III randomized controlled trial (RCT) has yet established clinical efficacy for any marine polysaccharide in digestive tract oncology. Future trials should prioritize chemically characterized preparations and pharmacokinetically informed dosing.

Regioselective modification enables precise regulation of polysaccharide activity. However, combined effects of multiple factors have not yet formed systematic structure–activity rules. These factors include reaction sites, degree of substitution, MW, and glycan linkage mode. This highlights the need to construct highly active polysaccharide derivatives through synthetic biology and directional modification. Such approaches clarify structure–activity relationships [181]. This solution responds to the structure–activity relationship gap. Marine polysaccharide bioactivity is determined by sulfation patterns, MW, and glycan conformation [17]. However, chemical modification strategies introduce safety considerations that warrant greater attention. Sulfation could raise clinically significant concerns over bleeding risk in cancer patients receiving anticoagulation therapy or undergoing surgery [182]. The degree of sulfation that optimizes anti-tumor immunomodulatory activity while maintaining an acceptable safety profile remains undefined. Beyond anticoagulation, high SPs risk excessive innate immune activation, including complement dysregulation and pro-inflammatory cytokine release [79]. Critically, long-term safety data encompassing chronic toxicity, potential immunosuppressive rebound, and microbiota impacts remain largely absent; this is a critical gap that regulatory agencies will be required to address before advanced clinical development can take place. In terms of delivery system design, various intelligent oral delivery systems responsive to intestinal microbiota have been developed recently. For instance, a colon-specific nanodelivery system maintains stability in the upper gastrointestinal tract. This system is constructed by covalently binding amifostine to pectin and encapsulating it with chitosan. It triggers drug release in response to pH changes and microbiota enzymatic hydrolysis in the colonic environment. This achieves precise delivery [183]. This advancement directly addresses low oral bioavailability and poor targeting of marine polysaccharides. It also enhances microbiota immune regulatory activity [141,171].

Realizing the translational potential of marine polysaccharides in digestive tract oncology requires coordinated progress across standardization, clinical trial design, and regulatory strategy. Batch-to-batch structural variability in fucoidan and carrageenan preparations represents a direct regulatory barrier; establishing pharmacopeial-grade standards with defined MW ranges, sulfation degrees, and monosaccharide composition, analogous to LMW heparin precedents, is the foundational prerequisite for clinical advancement. Biomarker-guided patient stratification, incorporating baseline gut microbiota composition, tumor P-selectin expression, and microsatellite instability status, will enable precision-guided trial enrollment. Near-term clinical priorities should include Phase I/II dose-escalation studies in Microsatellite Stable (MSS) colorectal cancer with SCFA profiling as the pharmacodynamic endpoint, window-of-opportunity surgical studies assessing tumor immune infiltrate remodeling, and combination trials pairing the pretreatment of marine polysaccharides with anti-PD-1/PD-L1 checkpoint inhibitors. Good Manufacturing Practice (GMP)-compliant and comprehensive preclinical safety packages will be required to satisfy regulatory agencies for oncological indications.

Marine polysaccharides exhibit unique advantages in remodeling the immune microenvironment of digestive tract tumors. They enhance anti-tumor immune responses by directionally regulating the gut microbiota–immune axis. This advantage integrates core findings of the entire review. These include bidirectional regulation of the gut microbiota immune axis in tumor progression, the flora-regulating and immunomodulatory effects of various marine polysaccharides, and their multi-level tumor inhibitory mechanism. However, their complex structural characteristics also pose challenges for the elucidation of structure–activity relationships and functional controllability, which echoes the key gaps summarized in the Introduction. Combining the study of marine polysaccharide structure–activity relationships with nanodelivery technology can overcome limitations. These limitations involve in vivo stability and targeting. This combination is a key pathway to promote translation from basic research to clinical applications. It closes the loop with core research objectives proposed in the Introduction. These objectives involve synthesizing research on marine polysaccharides regulating the gut microbiota–immune axis in digestive tract tumors and guiding their clinical translation.

In the future, based on interdisciplinary collaboration, integrating multi-omics analysis, synthetic biology, and nanodelivery strategies will facilitate the full exploration of the potential of marine polysaccharides to treat digestive tract tumors, providing a clear direction for addressing existing research gaps and advancing the clinical application of marine polysaccharides in tumor immunotherapy.

## Figures and Tables

**Table 1:** Representative marine polysaccharides regulating the gut microbiota–immune axis in digestive tract tumors.

Source	Polysaccharide	Specific Source	Key Targets	Biological Effects	References
Algae	Fucoidan	Brown algae	*H. pylori*, *Bifidobacterium*, and *Lactobacillus*	Selectively enriches beneficial bacteria, inhibits *H. pylori* adhesion, activates macrophages, and modulates TLR4/NF-κB signaling	[52,53,54]
	Alginate	*Laminaria japonica*	Gut microbiota and intestinal epithelial cells	Fermented to SCFAs, repairs intestinal barrier via tight junction upregulation, and enhances NK cell activity indirectly	[55,56,57]
	Ulvan	Green algae	*Akkermansia muciniphila*, *Bifidobacterium*, *Lactobacillus*, and *Bacteroides*	Promotes beneficial bacteria proliferation, increases SCFA production, inhibits Desulfovibrio, and establishes anti-inflammatory metabolic environment	[58,59,60]
Animal	SCP	Sea cucumber body wall/intestine	Gut microbiota, arginine metabolism pathway, tryptophan metabolism pathway, and immune cells	Optimizes flora structure, regulates arginine/citrulline metabolism, regulates tryptophan metabolism via the IDO1/Kyn/AHR axis, enhances NO synthesis and immune regulation, and prevents immunosuppressive microenvironment formation	[14,61,62,63,64,65]
	Chitosan	Shrimp and crab shells	*Lactobacillus*, *Bifidobacterium*, pathogenic bacteria, and DCs	Selectively promotes beneficial bacteria, inhibits pathogens via polycationic mechanism, and activates DCs via TLR4/cGAS-STING	[66,67,68,69]
Microorganism	Marine bacterial EPS	*Vibrio*, *Pseudomonas*, *Bacillus*, and *Aureobasidium*	*Pseudomonas aeruginosa*, *Vibrio harveyi*, gut microbiota, macrophages, GPX4, glutamate metabolism, and TCA cycle	Inhibits pathogen colonization, regulates GPX4 expression and GSH redox status, modulates ferroptosis-related immune processes via glutamate metabolism and TCA cycle, and improves the immunosuppressive microenvironment	[70,71,72,73,74]
	Marine fungal polysaccharide	*Aspergillus pseudoglaucus*, and *Aspergillus medius*	Gut microbiota, macrophages, T cells, and NK cells	Regulates amino acid metabolism, induces ferroptosis via Nrf2/SLC7A11/GPX4/ACSL4 axis, upregulates COX-2, iNOS and ROS, enhances immune cell function, and synergizes with PD-1/PD-L1 inhibitors	[75,76,77,78]

**Table 2:** Representative core mechanisms of marine polysaccharides regulating the gut microbiota–immune axis.

Mechanism Hierarchy	Target	Key Molecules	Functional Mechanism	References
Upstream mechanism (microbiota regulation)	Microbiota structure	*Firmicutes*/*Bacteroidetes* ratio, SCFA-mediated pH reduction	Enriches beneficial bacteria (*Lactobacillus*, *Akkermansia*, *Bifidobacterium*), restores *Firmicutes*/*Bacteroidetes* ratio, inhibits tumor-promoting microbiota	[83,84,85,86,87]
			Reduces intestinal pH, suppresses pathogen colonization	
	Metabolism	Microbiota fermentation enzymes	Promotes SCFA production, improves metabolic microenvironment, and reduces inflammation	[85,86,87]
		SCFA	Inhibits endotoxin release and reduces systemic inflammation	
	Barrier repair	Tight junction proteins	Upregulates tight junction proteins, maintains intestinal integrity, and blocks bacterial entry	[88]
		Epithelial barrier function	Reduces microbiota translocation and prevents bacterial dissemination	[88]
	Probiotic synergy	*Lactobacillus* colonization	Enhances *Lactobacillus* adhesion and colonization, and strengthens beneficial flora	[56]
Midstream mechanism (immune activation)	Innate immunity	GPR43, GPR41, CD80, CD86, and MHC-II	Activates M1 macrophage polarization via GPR43/41 and enhances phagocytosis and antigen presentation	[30,88,89,90]
			Promotes DC maturation and migration, improves T cell priming	
	Adaptive immunity	CD25, IFN-γ, TNF-α, HDAC, and mTOR	Induces CD8^+^ cytotoxic T cell proliferation via HDAC inhibition and mTOR activation, and enhances cytotoxic function	[91]
		HDAC, Foxp3, and H3 acetylation	Inhibits Treg differentiation via Foxp3 modulation, and suppresses immune tolerance	[29,32]
		PD-L1 and CTLA-4	Downregulates PD-L1 and CTLA-4, and restores T cell activity	[34,79]
	Inflammation	TNF-α, IL-6, and NF-κB	Suppresses TNF-α and IL-6 via NF-κB inhibition, and reduces tumor-promoting inflammation	[92,93]
		IL-12 and IFN-γ	Upregulates IL-12 and IFN-γ and promotes Th1 differentiation and NK cell activation	[29,94,95,96]
Downstream mechanism (tumor suppression)	Direct inhibition	Caspase-8, Bid, Bax, caspase-9, and PARP	Induces apoptosis via caspase-8 cascade and triggers mitochondrial apoptosis	[97]
		Cytochrome c, ERK, and GSK	Activates mitochondrial pathway and inhibits proliferation	[98]
		p53 and p38 MAPK	Bacterial polysaccharides: activates p53/p38 MAPK, induces cell-cycle arrest and apoptosis without toxicity to normal cells	[99]
	Indirect inhibition	HIF-1, VEGF, and PDGF	Inhibits HIF-1/VEGF/PDGF axis and vascular maturation and blocks hypoxia-driven angiogenesis	[100]
		VEGF, VEGFR-2, and Erk	Directly binds VEGF, blocks VEGFR-2/Erk signaling, and inhibits endothelial migration	[101]
		bFGF, PAI-1, and heparan sulfate	Regulates bFGF pathway, upregulates PAI-1, and blocks extracellular matrix degradation	[102]

**Table 3:** Structure–activity relationships of marine polysaccharides: impact of structural features on regulatory activity.

Structural Parameter	Parameter Characteristics	Impact on Microbiota	Impact on Immunomodulation	References
Molecular weight	Optimal range (5–50 kDa)	Readily fermented by probiotics; enhances intestinal barrier function	Facilitates PRR crosslinking; optimal receptor accessibility	[104,105,106,107]
	LMW (<10 kDa)	Facilitates probiotic fermentation	Facilitates endosomal escape; activates MyD88-dependent signaling; upregulates COX-2 and MCP-1; enhanced antioxidant capacity	[108,109]
	HMW (>50 kDa)	Increases luminal viscosity; forms physical protective barriers	Steric hindrance limits receptor accessibility; reduced direct immunostimulatory potency	[106,107,110,111]
Degree of sulfation	High sulfation	Activates macrophages via NF-κB and p38 MAPK; induces NO and cytokine production	Potent macrophage activation	[112,113]
	C2-sulfation	Generates axial negative charges; modulates bacterial recognition	Electrostatically engages TLR2/TLR4 co-receptors	[114]
	C4-sulfation	Shifts glycan conformation from gt to gg rotamers; optimizes binding to bacterial lectins	Not specified	[115,116]
	C2/C4 co-sulfation	3- to 5-fold-enhanced selectivity for *Bacteroides* over *Enterococcus*	Enhanced binding selectivity	[117]
Glycosidic linkage	β-(1→3) linkage	Extended helical conformation facilitates microbial recognition	Presents hydrophobic groove for Dectin-1 and TLR4/MD-2 recognition; triggers multivalent receptor clustering; activates ERK1/2 and p38 phosphorylation	[118,119,120,124]
	α-(1→4) linkage	Compact helical architecture limits microbial accessibility	Sterically occludes PRR-binding interfaces; attenuates immunostimulatory potency	[121]
Fucosylation pattern	Variable linkage type, branching density, and core structure	Modulates bacterial lectin recognition and mucosal colonization specificity	Regulates selectin binding and leukocyte homing	[122]
Uronic acid content	High iduronic acid	Guides species-specific microbial utilization; shapes community metabolic profiles	Activates complement system; strengthens innate immunity	[123]

## Data Availability

This review synthesizes findings and conclusions from previously published studies, all of which are fully cited in the reference list. The original data of the cited studies are accessible through their respective publications (e.g., main text) or public databases (as specified by the original authors). No new original data, including supplementary data such as literature screening records or data extraction tables, were generated in the preparation of this review.

## Literature Search Strategy

The electronic databases searched for this review include a comprehensive literature search of PubMed, Web of Science, and Scopus databases. The search scope covered publications from 2010 to 2026, with a focus on the literature published in the past decade. The search terms included combinations such as “marine polysaccharides”, “fucan”, “alginate”, “chitosan”, “gut microbiota”, “immunomodulation”, “immunomodulation”, “digestive tract tumors”, “polysaccharide structure–activity relationship”, and “nanodelivery systems”. By reviewing the reference lists of the articles included, other relevant references were also found. The studies were selected based on their relevance to the core themes of the review: the structural characteristics of marine polysaccharides, their regulatory effects on the gut microbiota and immune function, their therapeutic applications in digestive tract tumors, and the nanodelivery strategies of marine polysaccharides. The initial search yielded approximately 1000 records. After removing duplicates and filtering the titles and abstracts, a total of 165 references were finally included in this review. Additionally, an in-depth manual screening of the reference lists of the included articles was conducted to identify other relevant studies that were not covered by the database search.

## Abbreviations

The following abbreviations are used in this manuscript:

ACSL4	acyl-CoA synthetase long-chain family member 4
AF4	asymmetric flow field-flow fractionation
AHR	aryl hydrocarbon receptor
AKT	protein kinase B
AP-1	activator protein-1
Bax	Bcl-2-associated X protein
bFGF	basic fibroblast growth factor
Bid	Bcl-2 homology 3 interacting domain death agonist
BLIMP1	B lymphocyte-induced maturation protein 1
Caco-2	cancer coli-2
CagA	cytotoxin-associated gene A
CD25	cluster of differentiation 25
CD39	cluster of differentiation 39
CD44	cluster of differentiation 44
CD73	cluster of differentiation 73
CD8	cluster of differentiation 8
cGAS	cyclic GMP-AMP synthase
CLR	C-type lectin receptor
CoA	coenzyme A
COX-2	cyclooxygenase-2
CpG	cytosine–phosphate–guanosine
CTLA-4	cytotoxic T-lymphocyte-associated protein 4
DCs	dendritic cells
Dectin-1	dendritic-cell-associated C-type lectin-1
DS	degree of substitution
EPR	enhanced permeability and retention effect
EPSs	exopolysaccharides
ERK	extracellular signal-regulated kinase
Fap2	*Fusobacterium* adhesin protein 2
FOXP3	forkhead box P3
SF	sulfated fucoidan
FT-IR	Fourier transform infrared spectroscopy
Gal-GalNAc	galactose-N-acetyl galactosamine
GC-MS	gas chromatography–mass spectrometry
GILD	gastrointestinal and liver disease
GMP	good manufacturing practice
GPC	gel permeation chromatography
Gpr109a	G protein-coupled receptor 109A
GPR41	G protein-coupled receptor 41
GPR43	G protein-coupled receptor 43
GPR81	G protein-coupled receptor 81
GPX4	glutathione peroxidase 4
GSH	glutathione
GSK	glycogen synthase kinase-3
GSSG	glutathione disulfide
*H. pylori*	*Helicobacter pylori*
HDAC	histone deacetylases
HIF-1	hypoxia-inducible factor-1
HMA	humanized microbiota-associated
HMW	high molecular weight
HPAEC-PAD	high-performance anion-exchange chromatography with pulsed amperometric detection
HPGPC	high-performance gel permeation chromatography
IBD	inflammatory bowel disease
IDO	indoleamine 2,3-Dioxygenase
IFN-γ	interferon-γ
IgG	immunoglobulin G
IL-10	interleukin-10
IL-12	interleukin-12
IL-15	interleukin-15
IL-18	interleukin-18
IL-1β	interleukin-1β
IL-2	interleukin-2
IL-23	interleukin-23
IL-35	interleukin-35
IL-6	interleukin-6
iNOS	inducible nitric oxide synthase
IRAK	interleukin-1 receptor-associated kinase
ITIM	immunoreceptor tyrosine-based inhibitory motif
ITT	immunoglobulin tail-tyrosine motif-like domains
IκBα	inhibitor of κB alpha
Kyn	kynurenine
LAG3	lymphocyte activation gene-3
LC-MS	liquid chromatography–mass spectrometry
LJPS	Laminaria japonica polysaccharides
LMW	low molecular weight
LMWF-Lip	low-molecular-weight fucoidan-modified nanoliposomes
MAPK	mitogen-activated protein kinase
MCP-1	monocyte chemoattractant protein-1
MD-2	myeloid differentiation factor-2
MHC-II	major histocompatibility complex class II
MS	mass spectrometry
MSS	microsatellite stable
mTOR	mechanistic target of rapamycin
mTORC1	mammalian target of rapamycin complex 1
MW	molecular weight
MyD88	myeloid differentiation primary response 88
NF-κB	nuclear factor-kappa B
NK	natural killer cell
NLRC	NOD-like receptor family CARD domain-containing protein
NLRP3	NOD-like receptor family, pyrin-domain-containing 3
NMR	nuclear magnetic resonance
NO	nitric oxide
Nrf2	nuclear factor erythroid 2-related factor 2
PAI-1	plasminogen activator inhibitor-1
PARP	poly ADP-ribose polymerase
PD-1	programmed cell death protein 1
PDGF	platelet-derived growth factor
PD-L1	programmed death-ligand 1
PEG	polyethylene glycol
PI3K	phosphatidylinositol 3-kinase
PRR	pattern recognition receptor
RAS	rat sarcoma virus
RCT	randomized controlled trial
ROS	reactive oxygen species
S6K	ribosomal protein S6 kinase
SCFAs	short-chain fatty acids
SCPs	sea cucumber polysaccharides
SEC	size-exclusion chromatography
SLC7A11	solute carrier family 7 member 11
SMAD6	Sma- and Mad-related protein 6
SMAD7	Sma- and Mad-related protein 7
SPs	sulfated polysaccharide
STAT3	signal transducer and activator of transcription 3
STING	stimulator of interferon genes
Syk	Spleen tyrosine kinase
TAK1	TGF-β-activated kinase 1
TCA	tricarboxylic acid cycle
TGF-β	transforming growth factor-β
Th1	T helper 1 cells
Th17	T helper 17 cells
TIGIT	T cell immunoreceptor with Ig and ITIM domains
TIM3	T cell immunoglobulin and mucin-domain-containing-3
TLR4	Toll-like receptor 4
TLR9	Toll-like receptor 9
TME	tumor microenvironment
TNF-α	tumor necrosis factor-α
TRAF6	TNF receptor-associated factor 6
Treg	regulatory T cell
VEGF	vascular endothelial growth factor
VEGFR-2	VEGF receptor 2
YAP1	Yes-associated protein 1
ZO-1	zonula occludens-1
